# Clinical and Genetic Predictors of Glycemic Control and Weight Loss Response to Liraglutide in Patients with Type 2 Diabetes

**DOI:** 10.3390/jpm12030424

**Published:** 2022-03-09

**Authors:** Artemis Kyriakidou, Angeliki V. Kyriazou, Theocharis Koufakis, Yiannis Vasilopoulos, Maria Grammatiki, Xanthippi Tsekmekidou, Iakovos Avramidis, Stefanos Baltagiannis, Dimitrios G. Goulis, Pantelis Zebekakis, Kalliopi Kotsa

**Affiliations:** 1 Division of Endocrinology and Metabolism and Diabetes Center, First Department of Internal Medicine, Medical School, Aristotle University of Thessaloniki, AHEPA University Hospital, 1 St. Kiriakidi Street, 54636 Thessaloniki, Greece; artemiskir@gmail.com (A.K.); kelly__21.9@hotmail.com (A.V.K.); thkoyfak@hotmail.com (T.K.); grammatikimaria@gmail.com (M.G.); xanthippitsekmekidou@gmail.com (X.T.); pzempeka@auth.gr (P.Z.); 2 Department of Biology, Section of Genetics, Cell Biology and Development, University of Patras, 26500 Patras, Greece; iovasilop@upatras.gr; 3 Diabetes Center, Department of Internal Medicine, G. Papanikolaou General Hospital, 57010 Thessaloniki, Greece; iakovosavram@yahoo.gr; 4 Diabetes Outpatient Clinic, General Hospital of Kastoria, 52100 Kastoria, Greece; stefbalt@gmail.com; 5 Unit of Reproductive Endocrinology, First Department of Obstetrics and Gynecology, Medical School, Aristotle University of Thessaloniki, 56429 Thessaloniki, Greece; dgg@auth.gr

**Keywords:** type 2 diabetes mellitus, pharmacogenetics, GLP-1 RA, liraglutide, *CTRB1/2*, personalized medicine

## Abstract

Background: Evidence suggests a heterogeneous response to therapy with glucagon-like peptide-1 receptor agonists (GLP-1 RAs) in patients with type 2 diabetes mellitus (T2DM). The aim of this study is to identify the genetic and clinical factors that relate to glycemic control and weight loss response to liraglutide among patients with T2DM. Methods: The medical records of 116 adults with T2DM (51% female, mean body mass index 35.4 ± 6.4 kg/m^2^), who had been on treatment with liraglutide for at least 6 months and were genotyped for *CTRB1/2* rs7202877 (T > G) polymorphism, were evaluated. Clinical and laboratory parameters were measured at baseline, 3, and 6 months after initiating liraglutide treatment. The good glycemic response was defined as one of the following: (i) achievement of glycated hemoglobin (HbA_1c_) < 7% (ii) reduction of the baseline HbA_1c_ by ≥1%, and (iii) maintenance of HbA_1c_ < 7% that a patient already had before switching to liraglutide. Weight loss responders were defined as subjects who lost ≥3% of their baseline weight. Results: Minor allele frequency was 16%. Individuals were classified as glycemic control and weight loss responders (81 (70%) and 77 (66%), respectively). Carriers of the rs7202877 polymorphic allele had similar responses to liraglutide treatment in terms of glycemic control (odds ratio (OR): 1.25, 95% confidence interval (CI): 0.4, 3.8, *p* = 0.69) and weight loss (OR: 1.12, 95% CI: 0.4, 3.2, *p* = 0.84). In the multivariable analysis, higher baseline HbA1c (adjusted OR: 1.45, 95% CI: 1.05, 2.1, *p* = 0.04) and lower baseline weight (adjusted OR: 0.97, 95% CI: 0.94, 0.99, *p* = 0.01) were associated with better glycemic response to liraglutide, while higher baseline weight was associated with worse weight response (adjusted OR: 0.97, 95% CI: 0.95, 0.99, *p* = 0.02). Conclusions: Specific patient features can predict glycemic and weight loss response to liraglutide in individuals with T2DM.

## 1. Introduction

Glucagon-like peptide-1 receptor agonists (GLP-1 RAs) are a new class of drugs that have been recently added to the pharmacological armory against type 2 diabetes mellitus (T2DM). In addition to exhibiting strong glucose-lowering actions with minimal risk of causing hypoglycemia, they effectively reduce cardiovascular risk through their anti-atherosclerotic and anti-inflammatory properties. Furthermore, they promote weight loss and manifest favorable effects on common diabetes and obesity comorbidities, such as fatty liver [1].

Liraglutide is a long-acting human GLP-1 RA that is licensed for the management of T2DM in patients not adequately controlled with metformin [2]. In specific groups of patients with T2DM, such as those with established atherosclerotic disease or with multiple risk factors for CV disease, liraglutide can be used regardless of background therapy or quality of glycemic control [3]. In the LEADER trial, liraglutide was associated with a lower risk of major adverse CV events, CV death, and death from any cause compared with placebo [4]. Moreover, it has been approved for the pharmacological management of overweight and obesity independently of the presence of diabetes since administration at the dose of 3 mg, in addition to lifestyle measures, has been linked to clinically meaningful reductions in body weight. Finally, therapy with liraglutide positively affects several risk factors for CV disease, including blood lipids, systolic blood pressure, and albuminuria [5]. Collectively, these properties render this agent a fundamental component of CV risk management in patients with T2DM.

Clinical studies and daily practice suggest inter-individual variability in response to treatment with GLP-1 RAs in terms of glycemic control and bodyweight reduction. Anthropometric characteristics, baseline glycemia level, and genetic variants have been identified as potential predictors of the response to incretin therapies [6,7]. Therapeutic targets of HbA_1c_ following liraglutide treatment are achieved in T2DM patients with poorer baseline glycemic control [8,9]. Additionally, a greater reduction in HbA_1c_ has been reported in patients previously treated with monotherapy, particularly metformin [10]. Most studies have shown that a short duration of T2DM is associated with a better glycemic response to liraglutide [8,11], whereas glutamic acid decarboxylase antibodies and C-peptide concentrations <0.25 nmol/L are negative predictors [8]. The rs7202877 variant near *CTRB1* and *CTRB2* genes, which encode the digestive enzyme chymotrypsin, has been shown to be involved in the regulation of the incretin pathway, susceptibility to diabetes, and response to therapy with dipeptidyl-peptidase-4 inhibitors [12].

Despite the progress made in the pharmacotherapy of diabetes during the past years, a high percentage of people with T2DM are unable to meet therapeutic targets [13]. *Precision medicine* seeks to identify genetic, environmental, and lifestyle factors that could maximize the benefit of a specific intervention when applied to a person with certain characteristics. Considering the complex pathophysiology of T2DM and the wide variety of available therapeutic options, it is essential to recognize which treatment works best for which patient. Such a tailored approach in diabetes care could increase the proportion of patients capable of achieving glycemic and weight loss goals, thus, alleviating the complication burden for individuals, societies, and health care systems.

Clinical implications of the genetic polymorphism rs7202877 (T > G) in the *CTRB1/2* gene have not yet been extensively investigated. It has been hypothesized that this specific genetic variation might have an effect on the response to liraglutide treatment. The aim of the present study is to identify genetic and clinical factors related to glycemic control and weight reduction secondary to treatment with the GLP-1 RA liraglutide among patients with T2DM.

## 2. Materials and Methods

### 2.1. Study Population

The present retrospective observational cohort study evaluated the medical records of 200 patients with T2DM who were recruited from three Diabetes Centers in Greece: the Diabetes Center of the First Department of Internal Medicine of the Aristotle University in the AHEPA University Hospital of Thessaloniki, the Diabetes Center of the “G. Papanikolaou” General Hospital of Thessaloniki, and the Diabetes Outpatient Clinic of the General Hospital of Kastoria.

Patients were included in the study if they met all the following inclusion criteria: diagnosis of T2DM, established by the criteria of the American Diabetes Association (ADA) [14], aged ≥18 years, and being under treatment with liraglutide, either on 1.2 mg/day or 1.8 mg/day, for at least 6 months consecutively before the beginning of the study. Exclusion criteria were: diagnosis of another type of diabetes, treatment with GLP-1 RA other than liraglutide, and previous exposure to GLP-1 RA, and concurrent treatment with weight-reducing agents. Patients were also excluded in case the necessary clinical, laboratory, or genotypic data were not available at any time point during the study.

### 2.2. Study Protocol

Data on demographic characteristics, anthropometric measurements, and laboratory tests were obtained for each eligible participant at three time points: baseline (most recent value before liraglutide initiation) and 3 and 6 months after treatment initiation. Specifically, the collected data included gender, age, age at T2DM diagnosis, previous and concomitant treatment with hypoglycemic agents (number of agents used and drug class), weight, height, fasting plasma glucose (FPG), and glycated hemoglobin (HbA_1c_) concentrations. The genotype status of *CTRB1/2* rs7202877 of each participant was recorded. Body mass index (BMI) was calculated by dividing weight in kilograms by the square of height in meters, while the duration of diabetes by subtracting the age of T2DM diagnosis from the patient’s age. Previous treatment was divided into four categories: patients treated with oral hypoglycemic agents (OHAs), patients treated with OHAs and insulin, patients treated only with insulin, and patients with no previous treatment. OHAs included metformin, sulfonylureas, pioglitazone, DPP-4 inhibitors, sodium-glucose co-transporter-2 (SGLT-2) inhibitors, a fixed-dose combination of DPP-4 inhibitors and metformin, or a fixed-dose combination of SGLT-2 inhibitors and metformin.

Participants were initially classified into responders and non-responders to liraglutide treatment in terms of glycemic control. Responders referred to subjects who met at least one of the following criteria: (i) achievement of good glycemic control, defined as HbA_1c_ < 7% at either 3 or 6 months after treatment initiation, (ii) reduction of baseline HbA_1c_ levels by ≥1% after 3 or 6 months of use of liraglutide, (iii) maintenance of optimal glucose control (HbA_1c_ < 7%) that a patient had before switching to liraglutide after 3 or 6 months of treatment. Patients, who failed to fulfill any of those criteria were characterized as non-responders. The selection of response criteria was based on the critical evaluation of the National Institute of Health and Care Excellence (NICE) guidelines on the response to GLP-1 RA therapy [15] and the ADA clinical practice recommendations, suggesting that the HbA_1c_ target for most non-pregnant adults is <7% [16]. The achievement of this target and the 1% decrease in the HbA_1c_ value significantly reduce the risk of microvascular complications [16,17,18]. Subsequently, patients were further divided into two groups regarding their response to liraglutide treatment in terms of weight loss, independently of being or not responders to glycemic control. Responders were defined as subjects who lost ≥3% of their baseline weight, whereas non-responders were those who lost less than 3% of their initial weight after 3 or 6 months of liraglutide administration, according to the NICE definition of response to GLP-1 RA therapy [15].

### 2.3. Genotyping

Genotyping of the *CTRB1/2* rs7202877 polymorphism (T > G) was performed in the Laboratory of General Biology, Department of Medicine, University of Patras, Greece. Genomic DNA was obtained from venous blood samples of each patient, extracted and quantified, and candidate genotype analysis was conducted on stored DNA by established real-time polymerase chain reaction (real-time PCR) techniques. The protocol followed was the Real-Time PCR and Two-Step RT-PCR using Fluorescence Resonance Energy Transfer (FRET) Probes protocol using the QuantiFast Probe PCR + ROX Vial Kit (provided by QIAGEN^®^ Sample and Assay Technologies, Hilden, Germany). The real-time cycler used was the LightCycler^®^ 2.0 instrument (Roche, Basel, Switzerland). PCR was carried out with less than 200ng of genomic DNA, 0.6 μM of each oligonucleotide primer (primer forward: 5′-CACTATAAATGACCCAGTCTGAGGTA-3′ and primer reverse: 5′-CCTAAAAAACCAGAACTCTGCC-3′), 0.2 μM of each oligonucleotide probe (donor probe: 5′-ACGCCCTGACCGTCACAG-3′[Fluorescein] and acceptor probe: [LC-Red 640]5′-GACGCCCACTCGGAGTTCGGAGC-3′), 10 μL of 2x QuantiFast Probe PCR Master Mix (w/o ROX) with HotStarTaq Plus DNA polymerase (QIAGEN^®^), and RNase-free water in a final reaction volume of 20 μL. After activation of DNA polymerase by heating at 95 °C for 3 min, the PCR included DNA denaturation at 95 °C for 10 s, annealing at 50–60 °C for 15 s, and an extension at 72 °C for 15 s. This three-step process was repeated for 35–40 cycles.

### 2.4. Study Outcomes

The primary outcome of the study is to assess the association between the rs7202877 variant in *CTRB1/2* and the inter-individual variability of glycemic response to liraglutide treatment in Greek patients with T2DM. A secondary outcome was to evaluate the role of the polymorphism in predicting the weight loss response to liraglutide. Additionally, we aimed to examine potential clinical predictors of response to liraglutide, in terms of glycemia and weight control distinctly, by identifying differences in anthropometric and metabolic parameters between responders and non-responders.

### 2.5. Statistical Analysis

Statistical analysis was conducted for the entire cohort and each response outcome separately. Mean ± standard deviation (SD) or median and interquartile range (IQR) were used to describe central tendency and variation of quantitative variables, while frequencies were used to describe the distribution of categorical variables. Comparison of the genotype distribution between responders and non-responders was carried out by using logistic regression to calculate odds ratios (ORs) and 95% confidence intervals (CIs). Comparisons of quantitative variables between different groups were performed with Student’s *t*-test for parametric variables or with Mann–Whitney for non-parametric variables, while comparisons of categorical variables were conducted with the chi-square test or Fisher’s exact test. Repeated measures analysis of variance (ANOVA) or, in case of missing values, a mixed-effects model analysis was used to examine differences in weight, BMI, FPG, and HbA_1c_ over the three time points within each group. Pairwise post-hoc comparisons were performed using Tukey’s test. Predictive models were developed using logistic regression analysis for binary categorical dependent variables. Independent variables, which resulted in a *p*-value < 0.2 in the univariate analysis, were considered candidates for the multivariate model. A chi-square test was used to evaluate compliance of the genotype distribution to the Hardy–Weinberg equilibrium (HWE). The dominant genetic model was used in all analyses. Genotype TT was considered the first group, while combined TG and GG were considered the second group. All the *p*-values were two-tailed, and the alpha level of statistical significance was set at 0.05. All statistical analyses were conducted in the statistical software package RStudio (version 1.1.463, RStudio, Inc., Boston, MA, USA).

### 2.6. Ethical Considerations

The study was conducted in accordance with the principles of the Declaration of Helsinki and its later amendments. All study participants provided written informed consent before their enrollment in the study, and the study protocol was approved by the institutional review board of the Aristotle University of Thessaloniki (approval number 227/23 March 2016). 

## 3. Results

### 3.1. Participant Characteristics

Following the implementation of the eligibility criteria, the study population consisted of 116 individuals (51% females), with a mean age and BMI of 68.3 ± 10.9 years and 35.4 ± 6.4 kg/m^2^, respectively. Of participants, 78% were treated with OHAs before the initiation of liraglutide. Ninety-seven (84%) patients were homozygous for the wild-type rs7202877 T-allele (TT), and 19 (16%) carried one polymorphic G-allele (TG), while no patients carrying two polymorphic G-alleles were detected. The genotype distribution was in accordance with the HWE.

### 3.2. Glycemic Response to Liraglutide

In total, 81 (70%) patients were classified as glycemic responders and 35 (30%) as non-responders. The baseline characteristics of the two groups are presented in Table 1. Responders had a significant higher baseline HbA_1c_ value (mean difference: 0.6%, 95% CI: 0.2, 1, *p* = 0.007), and a significantly lower baseline weight (mean difference: −9.8 kg, 95% CI: −16.6, −2.9, *p* = 0.005) compared with non-responders.

The genotype distribution in relation to the response to treatment is shown in Table 2. Heterozygotes of the *CTRB1/2* rs7202877 variant had a similar glycemic response to patients homozygous for the wild-type (TT) (OR: 1.25, 95% CI: 0.4, 3.8, *p* = 0.69).

The anthropometric and metabolic characteristics of glycemic responders and non-responders at baseline, 3 and 6 months are shown in Table 3. Both groups demonstrated a significant reduction in weight and BMI from baseline to 6 months (*p* < 0.0001). Responders had significantly lower weight compared with non-responders at 3 (mean difference: −8.4 kg, 95% CI: −14.8, −2.1, *p* = 0.009) and 6 months (mean difference: −11.2 kg, 95% CI: −18.0, −4.4, *p* = 0.001).

Responders had lower FPG values at 3 (mean difference: −23 mg/dL, 95% CI: −39, −7, *p* = 0.006) and 6 months (mean difference: −19 mg/dL, 95% CI: −33, −6, *p* = 0.005) than non-responders. FPG was significantly reduced throughout the study in the response group (*p* < 0.0001), whereas no differences in FPG were observed in the non-response group (*p* = 0.73).

Responders had significantly higher HbA_1c_ before the initiation of liraglutide than non-responders. However, the former showed significantly lower HbA_1c_ concentrations at 3 (mean difference: −0.8%, 95% CI: −1.2, −0.3, *p* < 0.0001) and 6 months (mean difference: −1.2%, 95% CI: −1.5, −0.8, *p* < 0.0001) after the administration of liraglutide compared with the latter. Responders showed a significant reduction in HbA_1c_ from baseline to 3 months (mean difference: −1.2%, 95% CI: −1.5, −0.8, *p* < 0.0001) and from baseline to 6 months (mean difference: −1.3%, 95% CI: −1.7, −0.9, *p* < 0.0001), yet the change from 3 to 6 months was not significant (*p* = 0.19). Non-responders experienced a significant increase in HbA_1c_ values from baseline to 6 months (mean difference: 0.5%, 95% CI: 0.1, 0.8, *p* = 0.006).

In multivariate logistic regression (Table 4), the probability of being a glycemic responder to liraglutide was 45% higher when the baseline HbA_1c_ increased by 1% (adjusted for gender and baseline weight OR: 1.45, 95% CI: 1.05, 2.1, *p* = 0.04). Additionally, an increase in baseline weight by 10 kg was associated with a 26% reduced probability of being a responder (adjusted for gender and baseline HbA_1c_ OR: 0.97, 95% CI: 0.94, 0.99, *p* = 0.01). The gender did not show association with the response status in the multivariate model.

### 3.3. Weight Loss Response to Liraglutide

In total, 77 (66%) participants were classified as weight loss responders and 39 (34%) as non-responders. The baseline characteristics of the two groups are presented in Table 5. The baseline weight was significantly lower in the response group (mean difference: −7.8 kg, 95% CI: −14.5, −1.1, *p* = 0.02). Significantly more non-responders were treated with a combination of OHAs with insulin compared with responders (OR: 0.25, 95% CI: 0.07, 0.84, *p* = 0.01).

The genotype distribution in relation to weight loss response to treatment is shown in Table 6. The probability that a patient would be a weight responder is increased by 12% when the patient is a carrier of the polymorphic G-allele; however, the result was not significant (OR: 1.12, 95% CI: 0.4, 3.2, *p* = 0.84).

The anthropometric and metabolic characteristics of weight loss responders and non-responders at baseline, 3 and 6 months are shown in Table 7. Responders demonstrated a significant reduction in weight and BMI throughout the study period (*p* < 0.0001). Particularly, weight in the response group was reduced by 5.9 kg (95% CI: −6.9, −4.9, *p* < 0.0001) from baseline to 3 months and by 6.4 kg (95% CI: −8, −4.9, *p* < 0.0001) from baseline to 6 months. Following correction for multiple comparisons, the change of weight and BMI from 3 to 6 months in weight responders did not remain significant (*p* = 0.49 and *p* = 0.28, respectively). The overall changes in weight and BMI in the non-response group were not significant (*p* = 0.37 and *p* = 0.37, respectively). Responders maintained their lower baseline weight compared to non-responders at 3 (mean difference: −11.1 kg, 95% CI: −16.9, −5.2, *p* < 0.0001) and 6 months (mean difference: −13.3 kg, 95% CI: −19.9, −6.9, *p* < 0.0001). Although pretreatment BMI values did not differ between the two groups, responders had significantly lower BMI at 3 (mean difference: −2.4 kg/m^2^, 95% CI: −4.7, −0.2, *p* = 0.03) and 6 months (mean difference: −2.9 kg/m^2^, 95% CI: −5.3, −0.6, *p* = 0.01) compared with non-responders.

Responders presented a significant reduction in FPG from baseline to 3 months (mean difference: −27 mg/dL, 95% CI: −44, −9, *p* = 0.002) and from baseline to 6 months (mean difference: −28 mg/dL, 95% CI: −47, −9, *p* = 0.002) but not from 3 to 6 months (*p* = 0.94), while no significant differences in FPG were observed in the non-response group throughout the study period (*p* = 0.20).

Both weight responders and non-responders significantly reduced HbA_1c_ concentrations during the study (*p* < 0.0001 and *p* = 0.008, respectively). The reduction from baseline to 6 months was 0.9% (95% CI: −1.3, −0.4, *p* < 0.0001) for the response group and 0.5% (95% CI: −1, −0.1, *p* = 0.04) for the non-response group. However, the correction for multiple comparisons showed that changes in HbA_1c_ values from 3 to 6 months were not significant in both groups.

**Figure 1:**
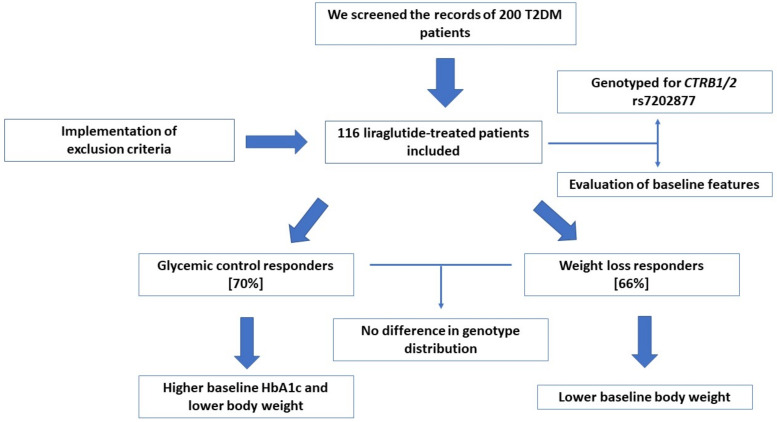
Methods and main findings of the study.

Baseline weight and HbA_1c_ were included in the multivariable predictive model (Table 8). Baseline HbA_1c_ was not significantly associated with the prediction of the weight response when adjusted for baseline weight. Moreover, the odds of a patient being classified as a weight-loss responder were decreased by 26% for every 10 kg increase in baseline weight (adjusted OR: 0.97, 95% CI: 0.95, 0.99, *p* = 0.02). Figure 1 illustrates the methods, outcomes, and main findings of the study.

## 4. Discussion

To our knowledge, this is the first study to investigate the association between gene polymorphisms and response to treatment with a GLP-1 RA in a Greek population. Among study subjects, 30% were classified as glycemic and 34% as weight loss non-responders to liraglutide administration. The proportions of patients who did not achieve the defined glycemic and weight goals coincide with the ranges of secondary failure to GLP-1 RA treatment observed in other studies [19,20,21,22], emphasizing the clinical importance of establishing predictors of response to therapy with these agents.

The carriers of the G-allele of rs7202877 in *CTRB1/2* did not exhibit a better response to treatment in terms of glycemic control and weight reduction. This finding is in line with the results of the study by T’Hart et al. [12], in which, although positive effects of the G-allele of rs7202877 on GLP-1-stimulated insulin secretion in individuals without diabetes were reported, no robust evidence to support the association between this genetic variant and response to GLP-1 RA treatment was provided. However, a worse treatment response was observed in G-allele carriers treated with DPP-4 inhibitors. Additional follow-up gene expression studies and function tests showed that rs7202877 acted as an expression quantitative trait locus for *CTRB1/2* and was associated with increased fecal chymotrypsin activity [12]. The *CTRB1/2* gene encodes chymotrypsinogen B1 and B2, which are secreted by the pancreas in the gut and are inactive precursors of the proteolytic enzyme chymotrypsin [12]. The understanding of the potential molecular mechanisms of actions related to rs7202877 is limited. It has been postulated that the enhanced pancreatic chymotrypsin expression and enzyme activity in the gastrointestinal tract, observed in G-allele carriers, leads to increased nutrient digestion, which might trigger alterations in L-cell stimulation and/or gastric emptying [12,23]. This hypothesized modulation of GLP-1 concentrations could improve incretin sensitivity of pancreatic β-cells and promote greater insulin secretion after an oral glucose load, thus lowering susceptibility to T2DM in G-allele carriers [24]. However, the fact that the rs7202877 variant has opposing effects on the risk for the two major types of diabetes [24,25] remains intriguing, as well as the divergent effects of this polymorphism on response to incretin-based therapies.

The current study demonstrates that the glycemic and weight loss response to liraglutide treatment are two distinct outcomes. Furthermore, the three time point analysis showed that the response, in terms of glycemic control and weight reduction, occurred primarily at 3 months. The same response was observed in another study, suggesting the use of the percentage change in HbA_1c_ after 3 months of exenatide treatment as a predictor of the response at 6 months [26]. Our findings are also in line with the analysis using pooled data from the SCALE Obesity and Prediabetes and SCALE Diabetes trials, which demonstrated that weight loss ≥4% at 16 weeks is an early response criterion for predicting ≥5% weight loss at 56 weeks in subjects treated with liraglutide 3 mg [27]. Glycemic responders had a higher HbA_1c_ value at baseline and achieved significantly lower HbA_1c_ during the study period compared with non-responders, with the reduction being more evident at the first 3 months after initiation of liraglutide. Both glycemic responders and non-responders achieved significant weight loss, indicating that liraglutide administration induced weight reduction independently of glycemic response. Regarding weight lowering response, patients classified as responders had significantly lower weight before treatment compared with non-responders, with a marked weight loss being manifested from baseline to 3 months. Since both weight responders and non-responders achieved HbA_1c_ concentrations <7% during the study, it is suggested that glycemic control was improved secondary to liraglutide treatment, irrespective of improvement in body weight.

Additionally, in the predictive model of glycemic response, higher HbA_1c_ and lower weight at baseline were associated with better treatment response. Baseline HbA_1c_ concentrations are the most reliable predictor of response to GLP-1 RA treatment, and most data report that the higher the baseline HbA_1c_, the higher the probability of good glycemic response [26,28,29,30]. Aroda et al. identified a group of liraglutide-treated patients with specific characteristics, namely, baseline HbA_1c_ < 8.5%, female sex, and short duration of diabetes (<5 years), who had the highest probability of achieving the composite outcome consisting of HbA_1c_ < 7%, no weight gain, and no hypoglycemia. These data underline the value of combining various patient features to predict the treatment response to GLP-1 RAs in general and to liraglutide in particular [31]. Preumont et al. [32] and Anichini et al. [33] exhibited opposing results in terms of pretreatment HbA_1c_ concentrations and response to GLP-1 RA, which could be attributed to the fact that both studies used the absolute reduction in HbA_1c_ to define a response. Insulin secretion and insulin resistance contribute to glycemic control [34]. In this context, a plausible explanation for the association observed in the present study could be that patients with higher baseline weight and, thus, a higher degree of insulin resistance tend to insufficiently stimulate insulin secretion after GLP-1 RA therapy and, therefore, exhibit poor response to treatment [35].

Regarding the prediction of the weight-lowering response, the results of other studies are conflicting; some of them showed that weight loss after GLP-1 RA treatment was greater in patients with a higher baseline BMI [30,36,37] while others reported no effect [21,38]. Haraguchi et al. [39] observed that poor responders had a higher baseline weight and BMI compared with strong responders, but the difference was not significant. However, most of those studies evaluated the response to exenatide. The greater proportion of patients treated with insulin before liraglutide initiation might be a reason for the higher baseline weight observed among weight non-responders [40,41].

The main strength of this study lies in the fact that it included a racially homogeneous population of Greek patients. This is important, given that interracial disparities have been postulated in the pathophysiology of diabetes and possibly in response to various glucose-lowering agents [42]. However, its findings should be interpreted in light of specific limitations. A larger sample size is needed to confirm the robustness of the association between the *CTRB1/2* rs7202877 polymorphism and the variability of response to liraglutide treatment. Additionally, the retrospective design of the study introduces selection and recall bias. Furthermore, the genotyping procedure was not further confirmed with sequencing. Participants were recruited from three different centers in Greece; hence, it was not feasible to minimize the variability of laboratory values due to differences in analytical procedures. Safety and adverse event outcomes were not included in the present analysis due to insufficient data. Finally, this work focused on a specific genetic variant due to technical and funding restrictions, whereas it is possible that many other polymorphisms in various genes are implicated in the therapeutic response to GLP-1 RAs. Despite its limitations, this study incorporated real-world data, providing clinicians with evidence that could be useful in the daily clinical setting.

## 5. Conclusions

Our findings indicate specific patient characteristics that could predict glycemic control and weight loss response to liraglutide in individuals with T2DM. In the era of personalized diabetes and obesity care, identifying such predictors could facilitate the achievement of glycemic and weight loss targets, thus mitigating the burden of complications from the disease.

## Figures and Tables

**Table 1:** Comparison of baseline characteristics between glycemic responders and non-responders.

	Glycemic Responders (*n* = 81)	Glycemic Non-Responders (*n* = 35)	*p*-Value
Age (years)	67.6 ± 10.8	69.7 ± 11.0	0.34
Gender (Female/Male)	44/37 (54/46)	15/20 (43/57)	0.26
Duration of T2DM (years)	15 (8.8)	16 (7.5)	0.17
Weight (kg)	92.9 ± 15.9	102.7 ± 19.5	0.01
Height (m)	1.64 ± 0.11	1.67 ± 0.08	0.18
BMI (kg/m^2^)	34.7 ± 6.2	36.9 ± 6.7	0.09
FPG (mg/dL)	169 ± 62	160 ± 54	0.45
HbA_1c_ (%)	7.9 ± 1.6	7.3 ± 0.7	0.01
Number of pretreatment agents	2.4 ± 1.1	2 ± 1	0.08
Pretreatment agents (%)			
OHAs	62 (77)	28 (80)	0.68
OHAs and insulin	11 (14)	5 (14)	1.00
Insulin	5 (6)	1 (3)	0.67
No treatment	3 (3)	1 (3)	1.00

Values are expressed as mean ± standard deviation (SD) for quantitative parametric variables, median (IQR) for quantitative non-parametric variables, and frequencies (percentage) for categorical variables. T2DM: Type 2 diabetes mellitus, BMI: body mass index, FPG: fasting plasma glucose, HbA_1c_: hemoglobin A_1c_, OHAs: oral hypoglycemic agents.

**Table 2:** Association between *CTRB1/2* rs7202877 polymorphism and glycemic response to liraglutide.

Genotype	Glycemic Responders (*n* = 81)	Glycemic Non-Responders (*n* = 35)	OR (95% CI)	*p*-Value
TG	14	5		
			1.25 (0.4, 3.8)	0.69
TT	67	30		

OR: odds ratio, CI: confidence interval.

**Table 3:** Clinical and laboratory parameters at baseline, 3, and 6 months after starting liraglutide treatment in glycemic responders and non-responders.

**Glycemic Responders (*n* = 81)**					**Adjusted *p*-Value**		
	**Baseline**	**3 Months**	**6 Months**	**Overall *p*-Value**	**Baseline to 3 Months**	**Baseline to 6 Months**	**3 Months to 6 Months**
Weight (kg)	92.9 ± 15.9	88.6 ± 14.9	87.9 ± 15.5	<0.0001	<0.0001	<0.0001	0.17
BMI (kg/m^2^)	34.7 ± 6.2	33.2 ± 5.6	32.8 ± 5.7	<0.0001	<0.0001	<0.0001	0.03
FPG (mg/dL)	169 ± 62	141 ± 35	136 ± 32	<0.0001	<0.0001	<0.0001	0.42
HbA_1_c (%)	7.9 ± 1.6	6.7 ± 1	6.6 ± 0.7	<0.0001	<0.0001	<0.0001	0.19
**Glycemic Non-Responders (*n* = 35)**					**Adjusted *p* Value**		
	**Baseline**	**3 Months**	**6 Months**	**Overall *p*-Value**	**Baseline to 3 Months**	**Baseline to 6 Months**	**3 Months to 6 Months**
Weight (kg)	102.7 ± 19.5 *	97.1 ± 15.9 *	99.1 ± 20 *	<0.0001	<0.0001	<0.0001	0.01
BMI (kg/m^2^)	36.9 ± 6.7	35.1 ± 5.7	35.6 ± 6.6 *	<0.0001	<0.0001	<0.0001	0.03
FPG (mg/dL)	160 ± 54	164 ± 42 *	156 ± 34 *	0.73	0.90	0.84	0.64
HbA_1c_ (%)	7.3 ± 0.7 *	7.5 ± 0.8 *	7.8 ± 1 *	0.006	0.20	0.01	0.24

Values are expressed as mean ± standard deviation (SD). The overall *p*-value shows the significance before adjustment, and the adjusted *p*-value shows the significance between time points after Tukey’s test for multiple comparisons. BMI: body mass index, FPG: fasting plasma glucose, HbA_1c_: hemoglobin A_1c_. * *p* < 0.05 for comparisons in different response groups at the same time point.

**Table 4:** Results from univariate and multivariable logistic regression, exploring variables that can predict glycemic response to liraglutide treatment.

	Univariate Analysis		Multivariable Analysis	
Variable	OR (95% CI)	*p*-Value	OR (95% CI)	Adjusted *p*-Value
Age (years)	0.98 (0.95, 1.02)	0.34		
Gender *	0.63 (0.28, 1.4)	0.20	0.73 (0.3, 1.7)	0.46
Duration of T2DM (years)	0.99 (0.95, 1.04)	0.73		
Genotype **	1.25 (0.41, 3.8)	0.69		
Baseline weight (kg)	0.97 (0.94, 0.99)	0.008	0.97 (0.94, 0.99)	0.01
Baseline BMI (kg/m^2^)	0.95 (0.89, 1.01)	0.09		
Baseline FPG (mg/dL)	1 (0.99, 1.01)	0.45		
Baseline HbA1c (%)	1.43 (1, 2.04)	0.04	1.45 (1.05, 2.1)	0.04
Number of pretreatment agents	1.41 (0.94, 2.1)	0.09		
Pretreatment agents ***				
OHAs and insulin	0.99 (0.32, 3.13)	0.99		
Insulin	2.26 (0.25, 20.24)	0.47		
No treatment	1.35 (0.13, 13.6)	0.79		

* Female gender was considered as the reference category. ** Genotype TT was considered as the reference category. *** Pretreatment with OHAs was considered as the reference category. T2DM: Type 2 diabetes mellitus, BMI: body mass index, FPG: fasting plasma glucose, HbA1c: hemoglobin A1c, OHAs: oral hypoglycemic agents. OR: odds ratio, CI: confidence interval.

**Table 5:** Comparison of baseline characteristics between weight loss responders and non-responders.

	Weight Loss Responders (*n* = 77)	Weight Loss Non-Responders (*n* = 39)	*p*-Value
Age (years)	68.5 ± 11	67.9 ± 10.6	0.77
Gender (Female/Male)	41/36 (53/47)	18/21 (46/54)	0.47
Duration of T2DM (years)	16 (9)	15 (10)	0.79
Weight (kg)	93.2 ± 16.3	101 ± 19	0.02
Height (m)	1.64 ± 0.1	1.68 ± 0.1	0.03
BMI (kg/m^2^)	35.1 ± 6.5	35.9 ± 6.3	0.51
FPG (mg/dL)	170 ± 63	158 ± 52	0.35
HbA1c (%)	7.8 ± 1.6	7.4 ± 1.1	0.17
Number of pretreatment agents	2.3 ± 1	2.2 ± 1.1	0.93
Pretreatment agents (%)			
OHAs	62 (81)	28 (72)	0.29
OHAs and insulin	6 (8)	10(26)	0.01
Insulin	5 (6)	1 (2)	0.66
No treatment	4 (5)	0 (0)	0.29

Values are expressed as mean ± SD for quantitative parametric variables, median (IQR) for quantitative non-parametric variables, and frequencies (percentage) for categorical variables. T2DM: Type 2 diabetes mellitus, BMI: body mass index, FPG: fasting plasma glucose, HbA_1c_: hemoglobin A_1c_, OHAs: oral hypoglycemic agents.

**Table 6:** Association between *CTRB1/2* rs7202877 polymorphism and weight loss response to liraglutide.

Genotype	Weight Loss Responders (*n* = 77)	Weight Loss Non-Responders (*n* = 39)	OR (95% CI)	*p*-Value
TG	13	6		
			1.12 (0.4, 3.2)	0.84
TT	64	33		

OR: odds ratio, CI: confidence interval.

**Table 7:** Clinical and laboratory parameters at baseline, 3, and 6 months after starting liraglutide treatment in weight loss responders and non-responders.

**Weight Loss Responders (*n* = 77)**					**Adjusted *p*-Value**		
	**Baseline**	**3 Months**	**6 Months**	**Overall *p*-Value**	**Baseline to 3 Months**	**Baseline to 6 Months**	3 **Months to 6 Months**
Weight (kg)	93.2 ± 16.3	87.3 ± 13.9	86.8 ± 15.0	<0.0001	<0.0001	<0.0001	0.49
BMI (kg/m^2^)	35.1 ± 6.5	32.9 ± 5.4	32.7 ± 5.9	<0.0001	<0.0001	<0.0001	0.28
FPG (mg/dL)	170 ± 63	143 ± 36	141 ± 36	<0.0001	0.002	0.002	0.94
HbA_1c_ (%)	7.8 ± 1.6	6.9 ± 1.2	6.9 ± 1.0	<0.0001	<0.0001	<0.0001	1.00
**Weight Loss Non-Responders (*n* = 39)**					**Adjusted *p*-Value**		
	**Baseline**	**3 Months**	**6 Months**	**Overall *p*-Value**	**Baseline to 3 Months**	**Baseline to 6 Months**	**3 Months to 6 Months**
Weight (kg)	101 ± 19 *	98.4 ± 16.3 *	100.1 ± 19.2 *	0.37	<0.0001	0.03	<0.0001
BMI (kg/m^2^)	35.9 ± 6.3	35.3 ± 5.9 *	35.6 ± 6.2 *	0.37	<0.0001	0.03	0.09
FPG (mg/dL)	158 ± 52	158 ± 41	145 ± 30	0.20	0.99	0.08	0.11
HbA_1c_ (%)	7.4 ± 1.1	7 ± 0.7	6.9 ± 0.7	0.008	0.03	0.04	0.98

Values are expressed as mean ± standard deviation (SD). The overall *p*-value shows the significance before adjustment, and the adjusted *p*-value shows the significance between time points after Tukey’s test for multiple comparisons. BMI: body mass index, FPG: fasting plasma glucose, HbA_1c_: hemoglobin A1c. * *p* < 0.05 for comparisons in different response groups at the same time point.

**Table 8:** Results from univariate and multivariable logistic regression, exploring variables that can predict weight response to liraglutide treatment.

	Univariate Analysis		Multivariable Analysis	
Variable	OR (95% CI)	*p*-Value	OR (95% CI)	Adjusted *p*-Value
Age (years)	1 (0.97, 1.04)	0.77		
Gender *	0.75 (0.35, 1.63)	0.47		
Duration of T2DM (years)	0.99 (0.95, 1.04)	0.75		
Genotype **	1.12 (0.4, 3.2)	0.84		
Baseline weight (kg)	0.97 (0.95, 1)	0.02	0.97 (0.95, 0.99)	0.02
Baseline BMI (kg/m^2^)	0.98 (0.92, 1.04)	0.50		
Baseline FPG (mg/dL)	1 (0.99, 1.01)	0.35		
Baseline HbA1c (%)	1.23 (0.91, 1.67)	0.17	1.23 (0.91, 1.68)	0.18
Number of pretreatment agents	1.02 (0.7, 1.48)	0.93		
Pretreatment agents ***				
OHAs and insulin	0.27 (0.09, 0.82)	0.02		
Insulin	2.26 (0.25, 20.24)	0.47		
No treatment	7068356 (0, ∞)	0.99		

* Female gender was considered as the reference category. ** Genotype TT was considered as the reference category. *** Pretreatment with OHAs was considered as the reference category. T2DM: Type 2 diabetes mellitus, BMI: body mass index, FPG: fasting plasma glucose, HbA1c: hemoglobin A1c, OHAs: oral hypoglycemic agents. OR: odds ratio, CI: confidence interval.

## Data Availability

The data presented in the study are available on request from the corresponding author.
